# Efficient Clustering for Continuous Occupancy Mapping Using a Mixture of Gaussian Processes †

**DOI:** 10.3390/s22186832

**Published:** 2022-09-09

**Authors:** Soohwan Kim, Jonghyuk Kim

**Affiliations:** 1 Department of Artificial Intelligence Software Technology, Sunmoon University, 70, Sunmoon-ro 221 beon-gil, Tangjeong-myeon, Asan-si 31460, Chungcheongnam-do, Korea; 2 The Center of Excellence for Cybercrimes and Digital Forensics, Naif Arab University for Security Sciences, Riyadh 11452, Saudi Arabia

**Keywords:** continuous occupancy mapping, Gaussian process, Dirichlet process, line tracking

## Abstract

This paper proposes a novel method for occupancy map building using a mixture of Gaussian processes. Gaussian processes have proven to be highly flexible and accurate for a robotic occupancy mapping problem, yet the high computational complexity has been a critical barrier for large-scale applications. We consider clustering the data into small, manageable subsets and applying a mixture of Gaussian processes. One of the problems in clustering is that the number of groups is not known a priori, thus requiring inputs from experts. We propose two efficient clustering methods utilizing (1) a Dirichlet process and (2) geometrical information in the context of occupancy mapping. We will show that the Dirichlet process-based clustering can significantly speed up the training step of the Gaussian process and if geometrical features, such as line features, are available, they can further improve the clustering accuracy. We will provide simulation results, analyze the performance and demonstrate the benefits of the proposed methods.

## 1. Introduction

Mapping is one of the fundamental problems of mobile robots which have to navigate through unknown environments. However, generating accurate and reliable environmental maps remains challenging, both theoretically and practically.

*Occupancy grid maps* [1] have been one of the most popular maps in robotics as its map representation is mathematically simple and easy to implement. At the core of its simplicity exists a strong assumption that the occupancy of a grid cell is independent of those of neighboring cells, making it suitable for real-time processing as well as for three-dimensional extension such as *Octomap* [2]. Many researchers have tried to relax this strict independence assumption to enhance the accuracy further by capturing the spatial correlation between the grid cells, for example, by utilizing a forward sensor model [3]. Occupancy grid maps also require a prefixed resolution on discretized input domains, making them unable to predict continuous occupancies at arbitrary positions.

O’Callaghan et al. [4,5] have considered occupancy mapping as a classification problem and applied *Gaussian processes* (GP) [6]. By doing that, they can exploit the dependence of occupancy values between grid cells and expand the map into a continuous space. The computational complexity related to the matrix inversion during training and inference of Gaussian processes, however, is $O(n^{3}$), with *n* being the number of data points; thus, it is not scalable to large-scale datasets. Moreover, the hyper-parameters within a global GP typically struggle to deal with local variations of occupancy in environments.

In this paper, we adopt a divide-and-conquer approach in building occupancy maps inspired by *a mixture of Gaussian processes* [7]. The benefits of this approach are two-fold: first, thanks to the reduced size of clustered data, the computational complexity of building occupancy maps dramatically decreases; second, the local structure of the map can be captured by training the local data. A mixture of Gaussian processes has been extensively studied. For example, Ross et al. [8] applied a Dirichlet process mixture of Gaussian processes to learn and categorize lung disease utilizing experts input constraints. Görür and Rasmussen [9] accelerated the performance of a Dirichlet process mixture of Gaussian processes by exploiting the conjugacy of the base distribution of the prior. Following this line of research, we apply a Dirichlet process mixture of Gaussian processes to solve the robotic occupancy mapping problem and develop an efficient clustering utilizing the geometrical information of the environment.

The contributions of this paper are:

- Dirichlet process-based clustering is investigated for occupancy mapping which does not require a prior on the number of clusters;
- Geometric feature-based clustering utilizing line tracking is developed to enhance the accuracy;
- Clustering and mapping performances of both methods are compared and analyzed.

To the best of our knowledge, there has been no prior work on utilizing clustering in the context of Gaussian process-based occupancy mapping, and this work is the elaboration of our previous work on occupancy mapping [10,11].

**Figure 1:**
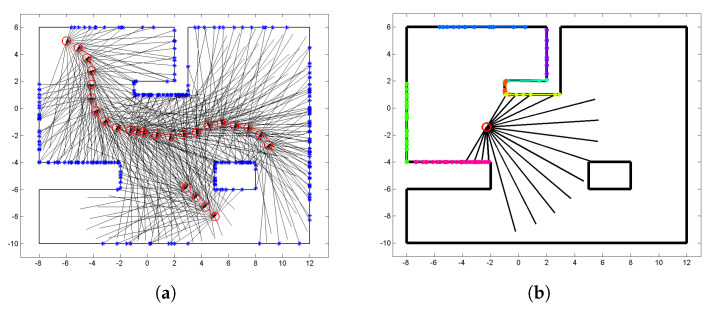
(**a**) Simulation data (Robots—red circles; laser beams—black lines; laser hit points—blue points). (**b**) Single laser scan (Laser hit points are grouped into clusters, with different colors per cluster).

Figure 1 illustrates a robotic mapping scenario used in this work. A robot equipped with a laser range finder explores the environment, while capturing laser scans. Since there is a limit on the measuring distance of the laser scanner, laser beams are observed with returns or with no returns. Our approach is not limited to a single-robot scenario. The goal is to estimate the continuous occupancy of the input space, by clustering laser sensor observations, training hyper-parameters of local Gaussian processes, and merging the local maps into a global occupancy map. The observation data comprise occupied and empty space—for example, the laser returns represent the occupied space, while the line segments the empty location.

The structure of the paper is as follows. In Section 2, we briefly review related work to our approach. We outline two clustering methods and three steps of our occupancy mapping in Section 3. In Section 4, experimental results are shown and the clustering and mapping performances are compared. We conclude the paper with future work in Section 6.

## 2. Related Work

A Gaussian process [6] is a Bayesian non-parametric approach to regression and classification and is widely used in robotics [12,13] and machine learning [14,15]. Lang et al. [16] and Hadsell et al. [17] have viewed building elevation maps as a regression problem and applied Gaussian processes. They are, however, unable to discriminate vertically overlapping objects such as tunnels and bridges as, basically, they estimate the height of the surface at each point of the ground, which are thus called 2.5D maps. For the full understanding of the structure of the environment, 3D occupancy or implicit surface representations are required [18,19].

Meanwhile, O’Callaghan et al. [4,5] applied Gaussian process classification to occupancy map building. They used laser hit points and discretized laser beams as measurements and stored laser hit points in a kd-tree. To reduce the size of data, they used the nearest points on laser beams to the query point. However, those training data are unique for each query point, and thus a new matrix must be generated and inverted each time a query point is evaluated. Later, they have addressed this problem by introducing the *integral kernel* [20] which integrated the point-wise kernel for laser beams. By doing that, they can reduce the size of data and reuse the same covariance matrix for every query point. However, the size of the training data acquired from large-scale environments is still too big to apply this approach.

In the machine learning community, the *mixture of experts* [21] scheme is commonly used as a divide-and-conquer strategy. With this concept, a *mixture of Gaussian processes* [7] has been proposed to reduce the size of the data and improve the performance. The number of Gaussian process experts, however, should be determined in advance, making the training process inflexible for other datasets.

We propose novel clustering methods to improve the accuracy and efficiency of Gaussian processes-based occupancy mapping. Notably, we utilize a geometrical feature extraction method, called line tracking, for efficient clustering and compare the performance with the Dirichlet process-based clustering method [10,11].

## 3. Building Occupancy Maps

**Figure 2:**
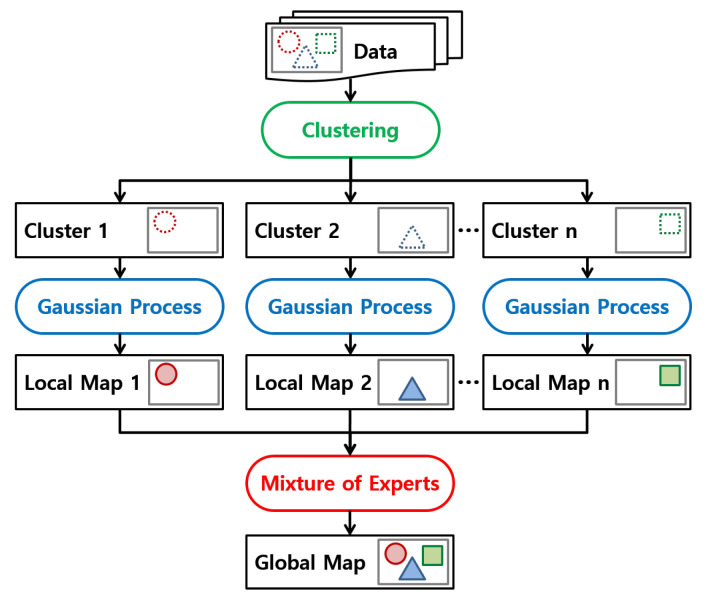
Flow chart of our mapping method using a Mixture of Gaussian processes. The inputs and outputs are conceptually visualized to show how data are clustered, how local maps are inferred from the clustered data, and how local maps are merged into a global map.

Our method for occupancy map building is composed of three steps which are specified in rounded rectangles in Figure 2. Since the size of the data is critical for kernel methods including Gaussian processes, we divide data into several clusters in the first step. Each Gaussian process is trained with its training subset and used to infer a local occupancy map at every query position in the second step. Finally, each local occupancy map is merged into one by using a mixture of Gaussian processes in the last step. Each step will be explained in detail in the following sections.

### 3.1. Data Clustering

#### 3.1.1. Dirichlet Process: DP-Clustering

The Dirichlet process (DP) is the infinite-dimensional generalization of a Dirichlet distribution. Similar to that, a Dirichlet distribution acts as a prior for a multinomial distribution, and works like a prior for an infinite-multinomial distribution. The major advantage of this method is that we do not need the number of clusters before clustering, as in *k*-means clustering.

Given *n* input data ${\left\{x_{i}\right\}}_{i=1}^{n}$, we assume that $x_{i}$ belongs to a cluster indicator $z_{i}$ and its distribution *F* is parameterized with $\theta_{i}$. Each parameter $\theta_{i}$ is drawn independently and identically from a discrete distribution *G* of a Dirichlet process,

(1) $$\begin{array}{cc} G & ∼\mathrm{DP}(\alpha ,G_{0}) \end{array}$$

(2) $$\begin{array}{cc} \theta & ∼G \end{array}$$

(3) $$\begin{array}{cc} x_{i}|z_{i},\theta & ∼F(\theta ), \end{array}$$

where α>0 is the concentration parameter which determines the variance of the Dirichlet process, and $G_{0}$ is the prior distribution over the component parameters θ.

The probability of assigning an input $x_{i}$ to either an existing component *z* or a new one $z_{new}$ given other part assignments $z_{/i}$, becomes [7]

(4) $$\begin{array}{cc} p(z_{i}=z|z_{/i}) & =\frac{n_{/i,z}}{n-1+\alpha }\\ p(z_{i}=z_{new}|z_{/i}) & =\frac{\alpha }{n-1+\alpha }, \end{array}$$

where $n_{/i,z}$ denotes the number of instances assigned to component *z* excluding $x_{i}$. Note that the number of clusters grows as the concentration parameter α increases.

Laser hit points are grouped via the Dirichlet process and then laser beam segments with returns are added to the same cluster of corresponding laser hit points. Laser beam segments with no returns are added to the clusters which have the most similar laser beam segments with returns. The similarity between laser beam segments can be determined using the *line integral kernel* of Equation (11). The main reason for this clustering process instead of clustering all together is to ensure that any cluster has at least one laser hit point. Notice that if a cluster has a laser hit point as a member, then the corresponding laser beam segment with the return is also included in the same group. In other words, we avoid those cases where a cluster is composed of only laser beam segments with no returns because we cannot train classifiers with input data of same target values.

#### 3.1.2. Line Tracking: LT-Clustering

Although the DP-clustering is highly flexible, it is prone to errors since the laser hit points are clustered based on the positions and underlying Gaussian distribution models. If the environment can be approximated by geometrical features such as lines or planes, they can be utilized for the clustering purpose. This observation inspired us to use geometric information when clustering data, and we propose to extract lines from laser hit points and cluster those on the same line into the same group. For 3D laser scan data, plane extraction methods can be applied correspondingly.

Among various line extraction methods, we choose *line tracking* (LT) [22], since it is known to outperform other methods. Algorithm 1 describes the procedure of line tracking [23] in which a line is fitted to laser hit points incrementally until it meets a point separated far enough.

After laser hit points are clustered along with extracted lines, similar to the DP-clustering, laser beams with returns are assigned to the same group of similar laser hit points. Laser beams with no returns are then allocated to the clusters which have the most similar laser beams with returns.
**Algorithm 1** Line trackingConstruct a line with first two points. **repeat**   **if** the next point is close to the line **then**     add it to the set of points and fit the line to them.   **else**     create a new line with the next two points.   **end if** **until** no points are remained

### 3.2. Local Occupancy Mapping

We assume that an occupancy map function f(x) of a position x follows a *Gaussian process* with the zero mean function and a covariance function $k(x,x^{\prime })$ as

(5) $$f\left(x\right)∼\mathrm{GP}\left(0,k(x,x^{\prime })\right),$$

where the *squared exponential* is adopted for the covariance function

(6) $$k\left(x,x^{\prime }\right)=\sigma_{f}^{2}\exp\left(-\frac{1}{2}\sum_{j=1}^{d}\frac{{\left(x_{j}-x_{j}^{\prime }\right)}^{2}}{l_{j}^{2}}\right).$$

Here, $\sigma_{f}^{2}$ represents the signal variance and $l={\left(l_{1},...,l_{d}\right)}^{⊤}$ denotes the length-scale in a *d*-dimensional space.

We also assume that the observations are associated with white noises,

(7) $$y=f\left(x\right)+\epsilon ,\;\epsilon ∼N\left(0,\sigma_{n}^{2}\right),$$

where $\sigma_{n}^{2}$ denotes the noise variance.

Then, given *n* noisy observations ${\left\{\left(x_{i},y_{i}\right)\right\}}_{i=1}^{n}$, the joint Gaussian distribution of the observed target values $y={\left(y_{1},...,y_{n}\right)}^{⊺}$ and a function value $f_{*}$ at a query point $x_{*}$ becomes

(8) $$\left[\begin{array}{c} y\\ f_{*} \end{array}\right]∼N\left(0,\left[\begin{array}{cc} K\left(X,X\right)+\sigma_{n}^{2}I & k(X,x_{*})\\ k{\left(X,x_{*}\right)}^{⊤} & k(x_{*},x_{*}) \end{array}\right]\right),$$

where $X={\left\{x_{i}\right\}}_{i=1}^{n}$, $K{\left(X,X^{\prime }\right)}_{i,j}=k\left(x_{i},x_{j}^{\prime }\right)$, and $k{\left(X,x_{*}\right)}_{i}=k\left(x_{i},x_{*}\right)$.

The conditional distribution of Equation (8) is also a Gaussian distribution as

(9) $$p\left(f_{*}|y\right)=N\left(f_{*};\mu_{*},\sigma_{*}^{2}\right).$$

The mean $\mu_{*}$ and covariance $\sigma_{*}^{2}$ are calculated in a closed form,

(10) $$\begin{array}{cc} \mu_{*} & =k_{*}^{⊤}{\left(K+\sigma_{n}^{2}I\right)}^{-1}y,\\ \sigma_{*}^{2} & =k_{**}-k_{*}^{⊤}{\left(K+\sigma_{n}^{2}I\right)}^{-1}k_{*}, \end{array}$$

where $K=K\left(X,X\right)\in R^{n\times n}$, $k_{*}=K\left(X,x_{*}\right)\in R^{n\times 1}$ and $k_{**}=k\left(x_{*},x_{*}\right)\in R$.

In classification as in occupancy mapping, the posterior becomes a non-Gaussian distribution, and the exact analytical inference becomes intractable. Approximation methods can be applied such as *Laplace approximation* [24] and *Expectation Propagation* [25]. In this work, we use Probabilistic Least Square Classification [26] and utilize a sigmoid function to squash the output values between 0 and 1 for binary classification.

In training, a laser hit point at position x is labelled as y=1 (occupied). The problem is the laser beams. Since the laser beams are continuous line segments, we need to discretize them into several points. However, this can cause a huge increase in the number of data points. Thus, we apply the *integral kernel* [20] which integrates the point-wise kernel $k(x,x^{\prime })$. With the integral kernel, a laser beam is considered as a data point and labelled as y=0 (unoccupied). The integral kernels for line-to-point and line-to-line similarity are

(11) $$\begin{array}{cc} k_{I}\left(l(u),x\right) & =\int_{0}^{1}k\left(l(u),x\right)du,\\ k_{II}\left(l\left(u\right),l^{\prime }\left(v\right)\right) & =\int_{0}^{1}\int_{0}^{1}k\left(l\left(u\right),l^{\prime }\left(v\right)\right)dudv, \end{array}$$

where l(u) and $l^{\prime }\left(v\right)$ are line segments parameterized with u,v∈[0,1]. In general, Equation (11) does not have a closed form formula and we applied *Simpson quadrature* [27] to numerically evaluate the integral. It should be mentioned that the concept of line kernel can be extended to area/volume kernel to further reduce the size of training data. With clustered data, the means and variances of the occupancy map functions in Equation (10) are predicted for each cluster, which correspond to local occupancy maps and map uncertainties.

### 3.3. Merging Local Occupancy Maps

The local maps need to be merged to provide global estimates at the query points. A mixture of experts model [21] merges local expert knowledge with corresponding weights,

(12) $$\mu_{*}=\sum_{i=1}^{k}p\left(z_{i}|x_{*},D\right)\mu_{*i},$$

where all the training data D={X,y} are split into *k* data sets $D=\{D_{1},...,D_{k}\}$. Here, the weight $p(z_{i}|x_{*},D)$ determines which of experts should be associated with which query points.

In this work we propose to use an approximation method, called a *Bayesian committee machine* [28], assuming each local map is independent given the query points and local clustered data. Then, Equation (12) can be rewritten as

(13) $$\begin{array}{cc} \frac{\mu_{*}}{\sigma_{*}^{2}}=\sum_{i=1}^{k}\frac{\mu_{*i}}{\sigma_{*i}^{2}}, & \;\frac{1}{\sigma_{*}^{2}}=\sum_{i=1}^{k}\frac{1}{\sigma_{*i}^{2}}. \end{array}$$

In this method, weighting factors are the normalized inverse variances. If a test position is close to some of the training data in one cluster, the variance would be small, and thus the effects of that group on the test area would be relatively higher than other groups. Therefore, we can avoid the accuracy loss problem of the distance-based gating network. Another benefit of this method is that the map uncertainty is also merged into one.

Please note that it is an approximation method assuming that each local map is independent of each other, conditioned on the local training data (rather than all training data), which is a reasonable assumption if each cluster contains a large enough training data set [28]. Moreover, the mixture of Gaussian processes [7] differs with our approach in that the former applies another Gaussian process to infer the probability that a query point belongs to each cluster. Therefore, it requires a global model which utilizes all the observations, which is thus not in agreement with our objective of divide-and-conquer.

### 3.4. Computational Complexity

Let us examine how much the computational complexity is reduced by introducing the mixture of Gaussian process to occupancy mapping. Suppose that the *n* training data is equally partitioned into *k* groups such that the number of data in each training subset becomes the same as $n_{i}=n/k$ for i=1,...,k. Since the computational complexity of a Gaussian process is $O(n^{3}+mn^{2})$, each Gaussian process expert costs $O({\left(n/k\right)}^{3}+m{\left(n/k\right)}^{2})$, and this is repeated *k* times for each cluster. Therefore, the total computational complexity of a mixture of Gaussian process experts is $O(\left(n^{3}+mn^{2}k\right)/k^{2})$. Recognize that with the global approximation, the large training data is partitioned into manageable subsets, and thus the cubic computational complexity with respect to the number of training data is dramatically reduced. Table 1 compares the theoretical complexity of the original Gaussian process and the mixture of Gaussian process experts.

## 4. Experimental Results

### 4.1. Simulation Data

Figure 1a shows the simulated data in which two robots are equipped with laser range-finders which sweep $180^{\circ }$ with 17 beams and whose maximum range is 8 m. The robots stop and acquire laser scans roughly at every half meter. Total 696 observations are obtained at 26 different poses in the environment of about 22 m × 18 m; 254 laser hit points, 254 laser beams with returns, and 188 laser beams with no returns.

### 4.2. Clustering Performance

**Figure 3:**
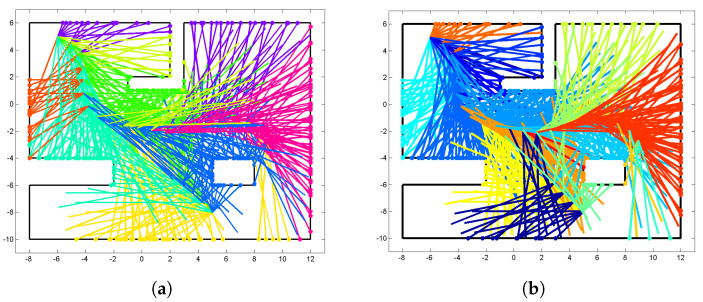
(**a**) Dirichlet process (DP)-based clustering with 8 groups. (**b**) Line tracking (LT)-based clustering with 18 groups.

Total 254 hit points are clustered into (1) 8 groups for the case of DP-clustering as shown in Figure 3a, and (2) 18 groups for LT-clustering as shown in Figure 3b, where hit points are color-coded for each cluster. The same number (254) of laser beams with returns are assigned to the groups where their end points (hit points) are located, while the other 188 laser beams with no returns are allocated to those clusters to which the most similar laser beams with return belonged based on the line-to-line integral kernel.

**Figure 4:**
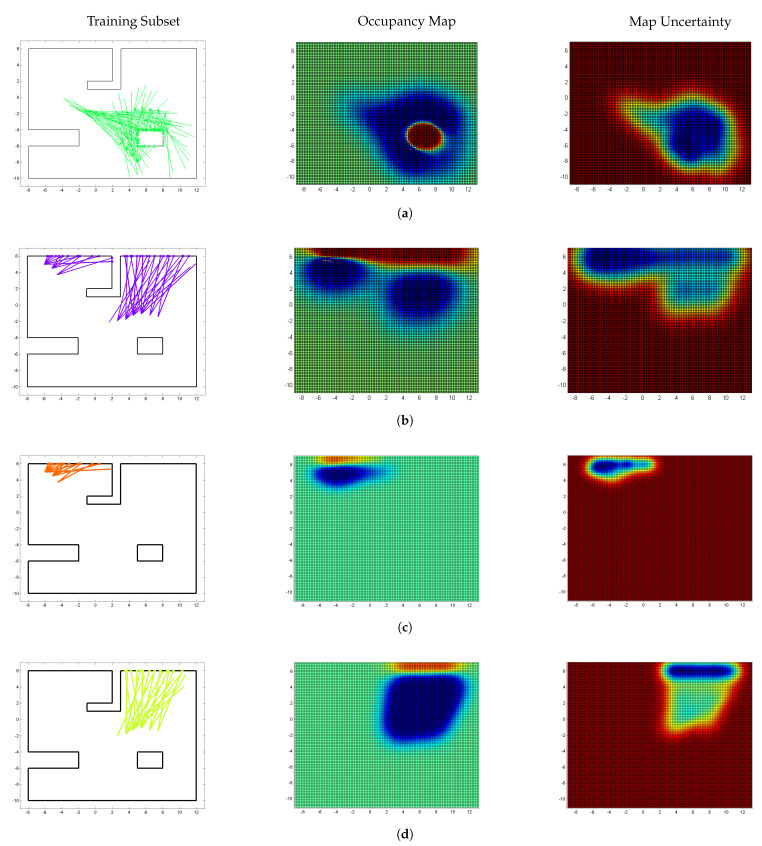
Occupancy maps and map uncertainties built with individual Gaussian process experts for training data partitioned with a Dirichlet process mixture model or the line tracking. (**left**) Training data subsets, (**middle**) Occupancy maps color-coded by occupancy (red/blue for occupied/empty), and (**right**) Map Uncertainties color-coded by uncertainty (red/blue for high/low uncertainty). (**a**) Cluster 4 using DP-clustering; (**b**) Cluster 5 using DP-clustering; (**c**) Cluster 3 using LT-clustering; (**d**) Cluster 13 using LT-clustering.

Figure 4 compares the clustering and related local mapping performance of two clustering methods; training data subsets (left column), continuous local occupancy maps (middle), and map uncertainties (right). Please note that each local Gaussian process predicted a local map covering the whole input space. Continuous occupancy maps correctly classified occupied and empty areas based on the clustered data. On the other hand, the map uncertainties are low where observations are made, and high where no observations are taken.

Figure 4a shows the mapping results of DP-clustering for Cluster-4, which correctly clustered data around the box at the bottom right corner. On the other hand, Figure 4b shows the mapping results of DP-clustering for Cluster-5, which incorrectly clustered data; the hit points from the two horizontal walls at the top of the environment are grouped in the same cluster. Consequently, the L-shaped wall between them is missing in the corresponding occupancy map with relatively high uncertainties. This prediction error is due to the clustering error. The Dirichlet process partitions data that explain the best of the base distribution, here a Gaussian distribution. Therefore, the hit points from the two horizontal walls at the top of the environment are clustered together with a horizontally thin Gaussian distribution. Mis-clustering leads to misprediction in local maps, thus causing errors in the final merged map.

In contrast, the LT-clustering successfully splits Cluster-5 into two separate groups. That is, Cluster-3 and Cluster-13, as shown in Figure 4c,d, respectively. Therefore, each wall is predicted separately, and the prediction errors in the previous method are rectified resulting in an improved performance.

**Figure 5:**
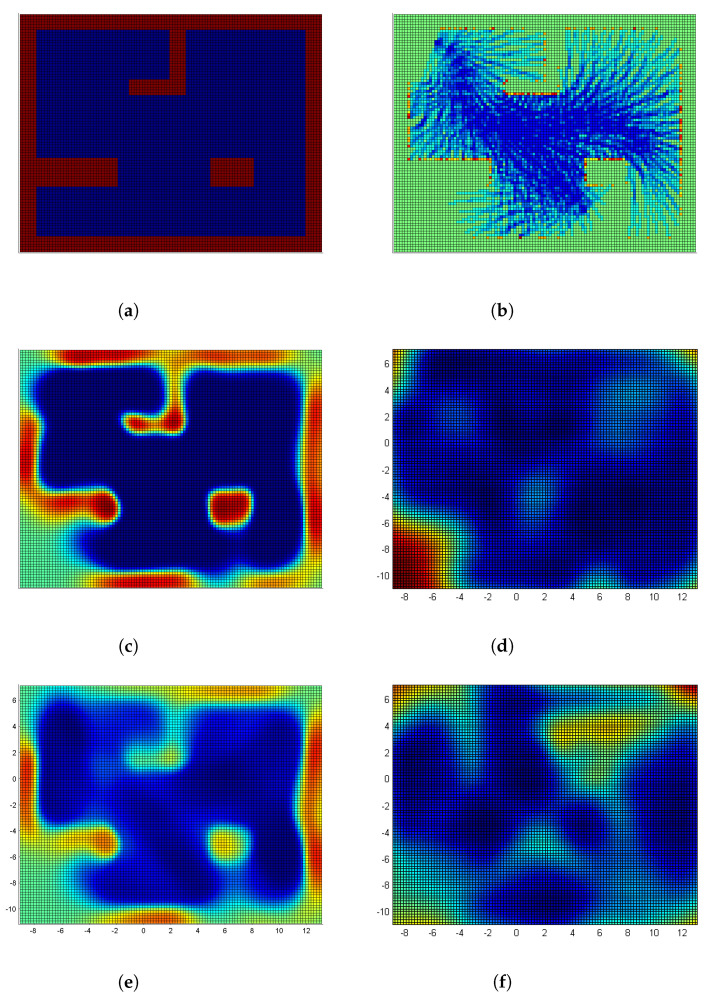

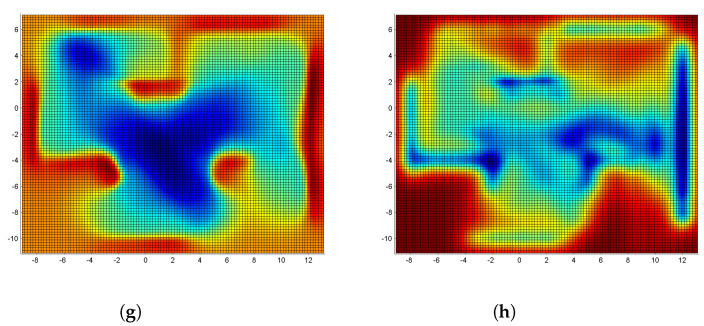
Comparison of occupancy maps between different approaches. (**a**) Simulation environment, where red and blue colors denote occupied and empty areas, respectively. (**b**) An occupancy grid map is discrete and sparse due to its independent cell assumption, while a Gaussian process generates (**c**) a continuous occupancy map with (**d**) its uncertainty from the same dataset, but suffers the high computational complexity. By utilizing a clustering method such as (**e**,**f**) a Dirichlet process (DP) and (**g**,**h**) line tracking (LT), we can reduce the computational complexity. However, a Dirichlet process only considers the distribution of points and may mis-cluster them, while the line tracking follows the connectivity of points and generates a better occupancy map with its uncertainty. (**a**) Ground Truth; (**b**) Occupancy Grid Map; (**c**) Occupancy Map (Single GP); (**d**) Map Uncertainty (Single GP); (**e**) Occupancy Map (DP-clustering); (**f**) Map Uncertainty (DP-clustering); (**g**) Occupancy Map (LT-clustering); (**h**) Map Uncertainty (LT-clustering).

Figure 5 compares occupancy maps and their uncertainties using various methods. Compared to the ground truth in Figure 5a, the occupancy grid map in Figure 5b gives a very accurate output. However, it is very sparse and no information is given regarding how much the output can be trusted. On the other hand, a single Gaussian process produces a continuous and accurate map with its uncertainty as shown in Figure 5c,d. However, due to its cubic complexity, the input data is clustered by a Dirichlet process in Figure 5e and by a line tracking in Figure 5g. Please note that the L-shaped wall from the final map of DP-clustering is less clear than that of LT-clustering showing the effect of the clustering errors.

**Figure 6:**
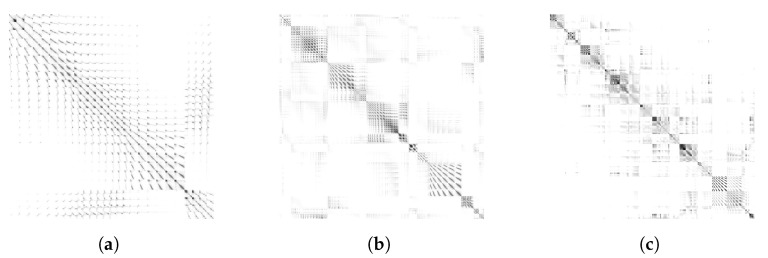
Covariance matrices between range observations (hit points, laser beams with returns and with no returns) constructed with integral kernels. Each element of the covariance matrix can be considered as pair-wise similarity between two observations where darker color shows higher similarity. (**a**) Covariance matrix of sequential observations before clustering. Nearby observations have high similarity, but some far observations also do. Repeated patterns are due to the laser beams spanning horizontally. The observations for the last several rows and columns are acquired from the second robot. After clustering using (**b**) a Dirichlet process (DP) and (**c**) line tracking (LT), the observations are grouped into diagonal blocks, which verifies that the clustering results are acceptable. (**a**) Before Clustering; (**b**) After DP-clustering; (**c**) After LT-clustering.

To analyze how well the observations (hit points, laser beams with returns and with no returns) are clustered, we further compare the covariance matrices constructed with the integral kernels before and after the clustering. The covariance matrix without clustering in Figure 6a depicts the pair-wise similarity between sequentially acquired observations which are ordered from left to right columns (also from top to bottom rows because it is symmetric). Nearby observations have relatively high similarity, but some departed observations also show some level of resemblance. Please note that the similarity increases and decreases periodically as the row and column indices increase, which is since the laser range finder keeps sweeping $180^{{}^{\circ}}$, thus re-observing the structures previously seen. Additionally, note that in the last few rows, a new similar pattern begins which is due to the observations acquired from the second robot starting at the bottom right of the environment.

After the clustering, the observations are successfully grouped, showing a block-diagonal pattern in the covariance matrices as shown in Figure 6b for DP-clustering and Figure 6c for LT-clustering, with a darker color representing higher similarity. In Figure 6b, dark rectangles are found along the diagonal, but there still exist off-diagonal correlations between clusters. These correlations occur because each region of groups is partially overlapped. Overall LT-clustering shows better clustering performance than DP-clustering, but with the cost of generating more clusters.

### 4.3. Map Accuracy

Now, the local Gaussian processes are merged for a global map, and we compare their results for two clustering methods as shown in Figure 5 with the ground truth data in Figure 5a. The normal occupancy grid map (OGM) in Figure 5b is sparse and fragmented. Particularly, the occupied areas on the walls are hardly estimated, which is mainly due to the static independence cell assumption of occupancy grid maps. Thus, only those grid cells which the laser beams pass through or return at are updated, and the others remain unknown.

On the other hand, the occupancy map built with a single Gaussian process shown in Figure 5c is more accurate and even dense due to the dependency assumption between Gaussian process outputs. Please note that even the occupancy values of unexplored areas are also estimated. However, as previously mentioned, the computational cost of this approach becomes too high, hence limiting its scalability.

The final map using DP-clustering is shown in Figure 5e, and is a little bit blurred compared to that of the single GP (Figure 5c). This blurring effect stems from the clustering errors, particularly around the L-shaped central area. Figure 5g,h show the occupancy map and its uncertainty using LT-clustering, respectively. Note that the occupancy map is also blurred, but the L-shaped wall is more clearly identified. Additionally, notice that the map uncertainty where the robot did not explore is high, while the map uncertainty along the robot trajectory is low.

**Figure 7:**
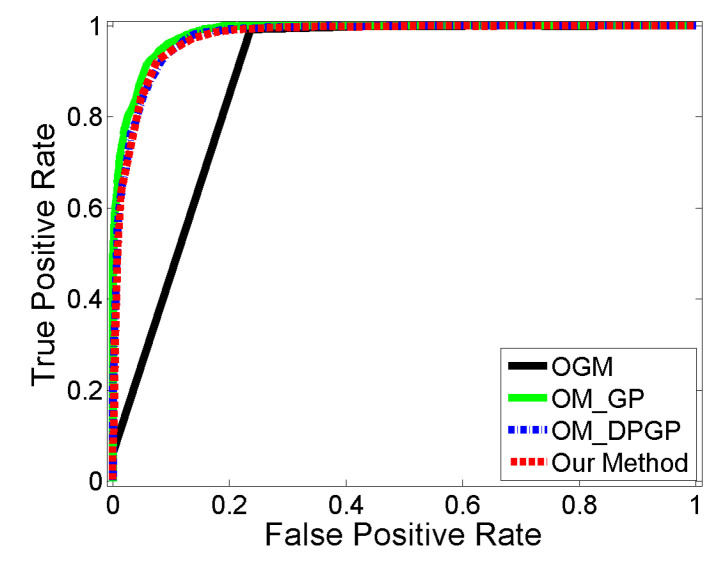
Receiver Operating Characteristic (ROC) of occupancy maps built by three different methods.

For a more exact comparison of accuracy, Receiver Operating Characteristic (ROC) curves for each method are drawn in Figure 7 showing that the performances of both clustering methods are similar each other and comparable to the single Gaussian process result.

### 4.4. Computational Time

To compare the computational complexity, we measure the computational time of learning and inference as shown in Table 2. Each method is implemented with Matlab and executed on a computer with an Intel Core 2 Duo 3.0 GHz CPU and 3.25 GB RAM.

OGM is the fastest since it does not require the training process. Note that the learning time of DP-clustering and LT-clustering method is dramatically reduced compared to the non-clustered approach. The method using LT-clustering shows a slightly faster result than that using DP-clustering. This result is because the line tracking creates more clusters than the Dirichlet process does, making the average group size smaller. However, their difference in inference time is negligible.

## 5. Discussion

We compare two clustering methods and analyze the experimental results more in depth. Since the map estimator is the same as a Gaussian process, and the local maps are merged by the mixture of experts as shown in Figure 2, the only thing that makes the final map different in Figure 5 is how the clustering is performed.

The DP-clustering only considers data points and their distributions. The number of clusters and cluster assignment is determined by how well the data points in each cluster explain the base distribution, i.e., a Gaussian distribution. Therefore, the points around the box at the bottom right corner are clustered in the same cluster with an almost isotropic Gaussian distribution as shown in Figure 4a, which produces a reasonable results. However, the points on the north wall are clustered in the same cluster with a almost flat Gaussian distribution as shown in Figure 4b, which generates a connected north wall without considering the L-shape wall in the middle. This mis-clustering caused errors when local maps are merged with a mixture of Gaussians.

On the other hand, the LT-clustering considers data points and their physical relations, i.e., the same wall of a line segment. Therefore, the points on the north wall are split into two different clusters as shown in Figure 4c,d. It is not shown, but the points around the box at the bottom right corner are split into four different clusters by line tracking. Therefore, we can say that physical relation-based or geometry-based clustering is a better choice for LiDAR data clustering than distribution-based clustering. Another benefit of line tracking is that the clusters can be determined incrementally along the data acquisition, which is crucial for online mapping strategy. However, a Dirichlet process assumes that all the data points are obtained before the clustering process begins. One drawback of LT-clustering is that, in general, a greater number of clusters are formed than DP-clustering, but that is a reasonable overhead to generate more accurate maps.

## 6. Conclusions

We have proposed two clustering methods for continuous occupancy mapping using Gaussian processes to improve the scalability and capturing local variations. We first presented Dirichlet process-based clustering which does not require prior knowledge on the number of groups. Although the computational complexity could be reduced significantly, it was prone to having clustering errors because it depends on the distribution of data points and resulted in a degraded mapping performance. On the other hand, if the environment contains some geometrical features, we showed that the line tracking-based clustering could be used more efficiently, improving the clustering performance while maintaining the mapping accuracy. One of the limitations of our approach is that the line tracking method can only be applied to 2D laser scan datasets. Therefore, applying plane extraction and tracking methods for 3D laser scan datasets will be our future work as well as a demonstration for large real datasets.

## Figures and Tables

**Table 1:** The comparison of computational complexities for a Gaussian process and its global approximation using a mixture of Gaussian process experts, where *n* and *m* denote the numbers of training and test data, respectively, and the training data are assumed to be equally partitioned into *k* subsets.

Gaussian Process	A Mixture of Gaussian Process Experts
$O(n^{3}+mn^{2})$	$O\left(\frac{n^{3}+mn^{2}k}{k^{2}}\right)$

**Table 2:** Computational time for learning and inference.

	Learning	Inference	Total
OGM	–	0.087 s	0.087 s
Single GP	11.9 h	103.0 s	11.9 h
Mixture of GPs with DP-clustering	1.5 h	17.2 s	1.5 h
Mixture of GPs with LT-clustering	1.4 h	11.6 s	1.4 h
